# Erythropoietin Receptor (EPOR) Signaling in the Osteoclast Lineage Contributes to EPO-Induced Bone Loss in Mice

**DOI:** 10.3390/ijms231912051

**Published:** 2022-10-10

**Authors:** Zamzam Awida, Sahar Hiram-Bab, Almog Bachar, Hussam Saed, Dan Zyc, Anton Gorodov, Nathalie Ben-Califa, Sewar Omari, Jana Omar, Liana Younis, Jennifer Ana Iden, Liad Graniewitz Visacovsky, Ida Gluzman, Tamar Liron, Bitya Raphael-Mizrahi, Albert Kolomansky, Martina Rauner, Ben Wielockx, Yankel Gabet, Drorit Neumann

**Affiliations:** 1 Department of Cell and Developmental Biology, Sackler Faculty of Medicine, Tel Aviv University, Tel Aviv 6997801, Israel; 2 Department of Anatomy and Anthropology, Sackler Faculty of Medicine, Tel Aviv University, Tel Aviv 6997801, Israel; 3 Department of Medicine A, Tel Aviv Sourasky Medical Center, Sackler Faculty of Medicine, Tel Aviv University, Tel Aviv 6423906, Israel; 4 Department of Medicine III & Center for Healthy Aging, Technische Universität Dresden, 01307 Dresden, Germany; 5 Institute for Clinical Chemistry and Laboratory Medicine, Technische Universität Dresden, 01307 Dresden, Germany

**Keywords:** erythropoietin (EPO), osteoclasts, erythropoietin receptor (EPOR), bone, CD115

## Abstract

Erythropoietin (EPO) is a pleiotropic cytokine that classically drives erythropoiesis but can also induce bone loss by decreasing bone formation and increasing resorption. Deletion of the EPO receptor (EPOR) on osteoblasts or B cells partially mitigates the skeletal effects of EPO, thereby implicating a contribution by EPOR on other cell lineages. This study was designed to define the role of monocyte EPOR in EPO-mediated bone loss, by using two mouse lines with conditional deletion of EPOR in the monocytic lineage. Low-dose EPO attenuated the reduction in bone volume (BV/TV) in Cx3cr1^Cre^ EPOR^f/f^ female mice (27.05%) compared to controls (39.26%), but the difference was not statistically significant. To validate these findings, we increased the EPO dose in LysM^Cre^ model mice, a model more commonly used to target preosteoclasts. There was a significant reduction in both the increase in the proportion of bone marrow preosteoclasts (CD115^+^) observed following high-dose EPO administration and the resulting bone loss in LysM^Cre^ EPOR^f/f^ female mice (44.46% reduction in BV/TV) as compared to controls (77.28%), without interference with the erythropoietic activity. Our data suggest that EPOR in the monocytic lineage is at least partially responsible for driving the effect of EPO on bone mass.

## 1. Introduction

The hormone erythropoietin (EPO) is produced predominantly by the fetal liver and adult kidney and is then released to the circulation to regulate red blood cell production [1]. EPO binding to the EPO receptor (EPOR) expressed on erythroid progenitors stimulates their survival, proliferation, and differentiation [2]. Deletion of EPO or EPOR in mice results in lethal embryonic anemia [1,3].

Clinically, recombinant human EPO (rHuEPO) is widely used to treat anemia in patients with advanced chronic kidney disease or that induced by cancer and chemotherapy, particularly in cases of multiple myeloma (MM) and myelodysplastic syndromes (MDS) [4,5,6,7].

EPOR has been detected on a wide variety of non-hematopoietic cells, such as neurons [8,9,10]; endothelial cells [11,12]; skeletal muscle cells [13,14]; various immune cells, including macrophages and dendritic cells [15]; adipocytes [16]; stromal cells [17]; preosteoclasts [18]; and osteoblasts [18,19,20,21,22,23]. This suggests that EPO activates EPOR in a variety of settings to elicit diverse biological responses that are unrelated to erythropoiesis.

The interaction between EPO and the skeletal system, and specifically with bone, has attracted special attention, since we and others have shown that high EPO levels in mice (either from endogenous overexpression or exogenous administration) lead to massive bone loss [17,18,21,22,24,25,26]. Accordingly, there are recent reports that high EPO levels are linked to reduced bone mass in humans [27,28,29,30]. Of note, the effects of EPO on bone are context- and dose-dependent [26,31], and, under certain conditions, EPO can also increase bone mass [32].

Bone remodeling is a consequence of the coordinated activities of monocyte-derived multinucleated osteoclasts, which mediate bone resorption, and mesenchymal stem cell (MSC)-derived osteoblasts, which mediate bone formation [33,34,35]. Whereas the osteoclast lineage directs inflammatory processes and bone resorption, the mesenchymal lineage is responsible for bone regeneration and immune modulation. Coupling factors are involved in this process, and some are osteoclast derived factors, including bone morphogenetic proteins (BMPs), Wnt10b, ephrinB2, and semaphorin-4D (sema4D), and some are osteoblast-derived, such as the receptor activator of nuclear factor kappa-B ligand (RANKL), osteoprotegerin (OPG), and macrophage colony stimulating factor (M-CSF) [36]. The role of MSCs within the bone marrow is not limited to their function as the progenitors of osteoblasts, as they also secrete biologically active molecules that stimulate tissue repair and modulate local immune response in a paracrine manner [37]. Among these factors, extracellular vesicles, and particularly the exosomes, have been reported to be therapeutically efficacious in bone regeneration [38,39].

We and others have previously demonstrated that EPO-induced bone loss in mice is mediated via action on osteoblasts [19,21,22] and B cells [40] in the bone marrow (BM) niche. However, the ablation of EPOR in these cell lineages did not completely abrogate the skeletal effect of EPO administration, suggesting the involvement of EPOR on additional cells. Recent results from our lab indicate that both high and physiologically relevant doses of EPO induce osteoclast differentiation in vitro by direct signaling through EPOR on osteoclast precursors [18,41,42]. This effect can be blocked by a specific non-erythropoietic EPO analog [42]. However, the question of whether EPOR on preosteoclasts directly contributes to EPO-driven bone loss in vivo has not yet been resolved.

This study was designed to assess the physiological and therapeutical roles of preosteoclast EPOR in the EPO-mediated bone resorption. For this purpose, we utilized the Cre/LoxP system to generate two separate mouse lines with non-overlapping off-target sites of conditional EPOR knockout in the monocyte/macrophage lineage. One model is based on the LysM^Cre^ construct, which targets the myeloid lineage, including macrophages and preosteoclasts, and is a well-established model for specific recombination starting in early osteoclast progenitors [43,44,45]. The second model uses the Cx3cr1^Cre^ construct, which is more specific to macrophages but is less commonly used to study osteoclast biology [46,47,48,49].

Herein, we present data suggesting that EPOR signaling in preosteoclasts contributes at least in part, to the observed EPO-mediated bone loss.

## 2. Results

### 2.1. The Skeletal Effect of EPOR Deletion in the Monocytic Lineage Using Cx3cr1^Cre^

**Figure 1:**
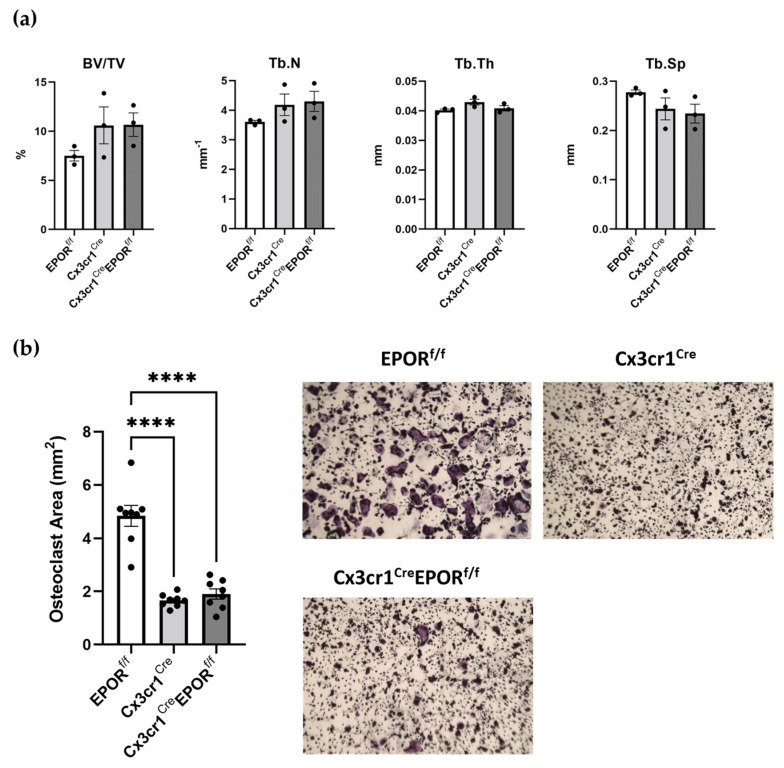
**Monocyte-specific EPOR knockout has no effect on trabecular bone mass.** (**a**) μCT analysis of the distal femoral metaphysis of 11-week old female transgenic mice carrying a conditional knockout of EPOR in the monocytic lineage (Cx3cr1^Cre^EPOR^f/f^) as compared to EPOR^f/f^ and Cx3cr1^Cre^ controls. n = 3 in each group. Trabecular bone volume/total volume (BV/TV, %); trabecular number (Tb.N, mm^−1^); trabecular thickness (Tb.Th, mm); trabecular separation (Tb.Sp, mm). (**b**) Total area of the multinucleated TRAP+ osteoclasts grown with M-CSF and RANKL in vitro from bone-marrow-derived macrophages isolated from female Cx3cr1^Cre^EPOR^f/f^ and their controls. Representative images acquired at ×2 magnification presented in the right panel. Cells were pooled from two mice per group, and eight replicates were prepared from each group. ****, *p* < 0.0001 versus EPOR^f/f^. All data are mean ± SEM. Data were analyzed by 1-way ANOVA.

We have generated a murine model of conditional EPOR knockout in the monocytic lineage, wherein the Cre-recombinase is driven by the Cx3cr1 promoter (Cx3cr1^Cre^ EPOR^f/f^). In a previous study, Cx3cr1-deficient mice displayed slight but significant increases in trabecular and cortical thickness and reduced numbers of osteoclasts compared to wild-type mice [50]. In line with this report, herein, we also detected a slight increase in trabecular bone parameters in Cx3cr1^Cre^ females (Figure 1a); although, possibly due to the small sample size, the difference did not reach statistical significance. Consistent with this bone phenotype, ex vivo osteoclastogenesis was also significantly reduced (~2.9 fold) in the Cx3cr1^Cre^ compared to the EPOR^f/f^ mice (Figure 1b). Importantly, to exclude the possibility that any of the skeletal phenotypes observed are due to the expression of the Cx3cr1^Cre^ transgene rather than DNA recombination in the flox EPOR, we used Cx3cr1^Cre^ animals as the control group for the Cx3cr1^Cre^ EPOR^f/f^ mice, as previously reported for other Cre transgenic mice [51,52].

The results revealed that EPOR deletion in the monocytic lineage is unlikely to affect the trabecular bone parameters or osteoclastogenesis in female mice (Figure 1a,b).

### 2.2. Confirmation of Conditional EPOR Deletion in the Monocytic Lineage

**Figure 2:**
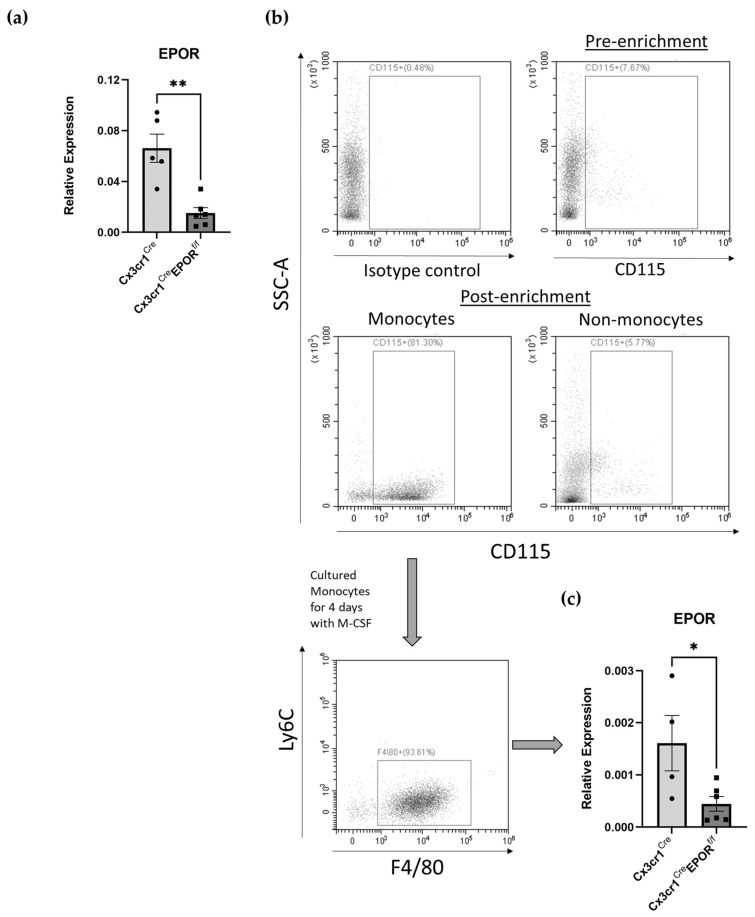
**Confirmation of the conditional knockout of EPOR expression in the monocytic lineage in the Cx3cr1^Cre^EPOR^f/f^ mice.** (**a**) EPOR expression as measured by RT-qPCR in freshly isolated bone-marrow-derived CD115^+^ cells from Cx3cr1^Cre^EPOR^f/f^ mice and their controls. Expression of EPOR was normalized to HPRT. n = 5–6 mice in each group. ** *p* < 0.01 vs. Cx3cr1^Cre^. (**b**) Flow cytometry analysis of freshly isolated BM monocytes after 4 days culture with M-CSF to generate BMDM in vitro. (**c**) EPOR expression as measured by RT-qPCR in BMDM as in (**b**) from Cx3cr1^Cre^EPOR^f/f^ mice and their controls. Expression of EPOR was normalized to HPRT. n = 4–6 mice in each group. * *p* < 0.05 vs. Cx3cr1^Cre^. All data are mean ± SEM. *p* values calculated by a Student’s *t*-test.

To validate the Cx3cr1^Cre^ EPOR^f/f^ model, bone marrow (BM)-derived CD115^+^ cells were isolated from 12-week-old male and female Cx3cr1^Cre^ EPOR^f/f^ mice and the appropriate controls, using a CD115 microbead kit, and the cells were then probed for the expression of EPOR (Figure 2a). In addition, isolated BM monocytes were differentiated into bone-marrow-derived macrophages (BMDM), which were then probed for the expression of EPOR (Figure 2b,c). The results presented in Figure 2a,c confirm a significant knockout of EPOR in the Cx3cr1^Cre^ EPOR^f/f^ mice versus controls, with a 5- and 3.5-fold reduction in EPOR mRNA levels in CD115^+^ cells and BMDM, respectively.

### 2.3. EPO Erythropoietic Activity Is Preserved in the Cx3cr1^Cre^ EPOR^f/f^ Mice

**Figure 3:**
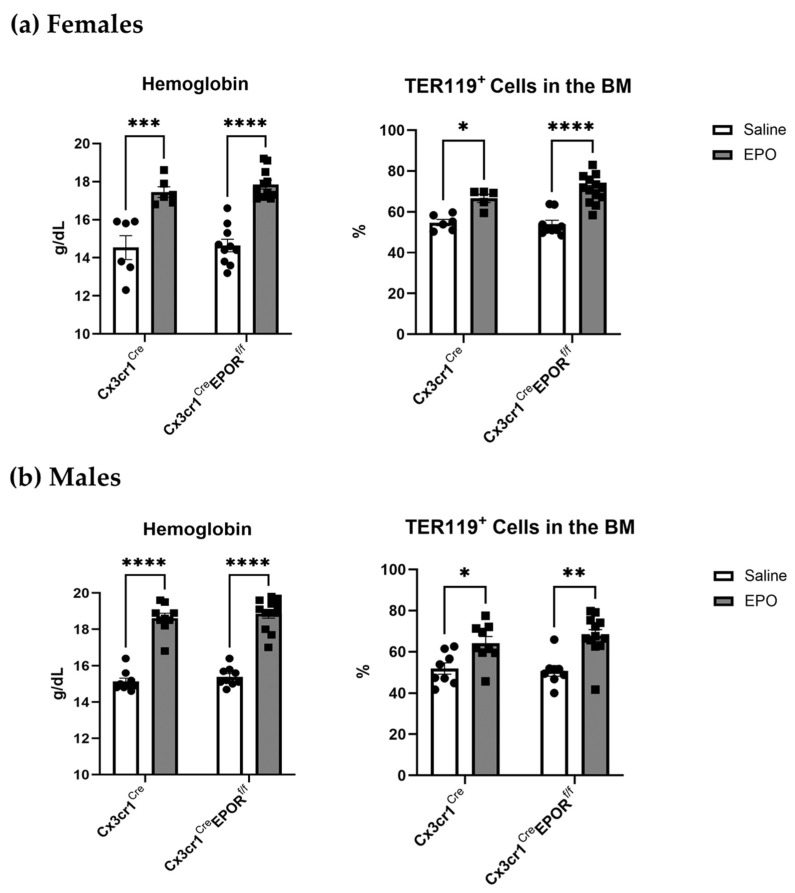
**EPOR knockout in the monocytic lineage does not interfere with the erythropoietic response to EPO.** Left panels, hemoglobin levels in Cx3cr1^Cre^EPOR^f/f^ as compared to their Cx3cr1^Cre^ controls after treatment with EPO (60 IUx3 per week for 2 weeks). Right panels, flow cytometry analysis of TER119^+^ erythroid progenitors in the bone marrow. (**a**) Females, (**b**) males. n = 6–12 mice in each group. * *p* < 0.05, ** *p* < 0.01, *** *p* < 0.001, and **** *p* < 0.0001. All data are mean ± SEM. *p* values were calculated by 2-way ANOVA.

To test the effect of monocytic EPOR deletion on the erythropoietic capacity of EPO, we measured the levels of hemoglobin and evaluated the numbers of TER119^+^ erythroid progenitor cells in the bone marrow by flow cytometry. As expected, EPO treatment resulted in a significant increase in these parameters, none of which were affected by EPOR knockout in the Cx3cr1 expressing cells (Figure 3).

### 2.4. Cx3cr1^Cre^ EPOR^f/f^ Mice Are Partially Protected against EPO-Induced Bone Loss

**Figure 4:**
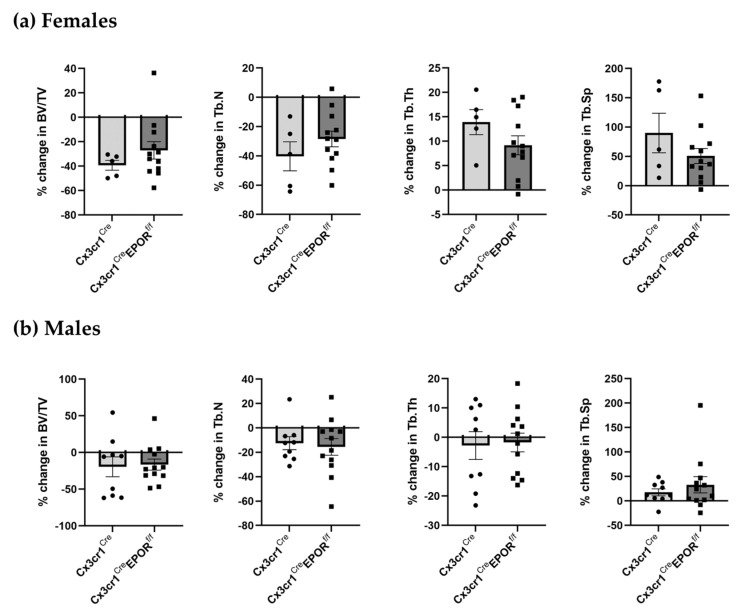
**EPOR deletion in the Cx3cr1-expressing monocytic lineage somewhat mitigates EPO-induced bone loss.** μCT analysis of the distal femoral metaphysis of saline- and EPO-injected 12-week-old (**a**) female (n = 5–12 in each group) and (**b**) male (n = 9–12 in each group) transgenic mice carrying a conditional knockout of EPOR in the monocytic lineage Cx3cr1^Cre^EPOR^f/f^ as compared to their Cx3cr1^Cre^ controls. Data are represented as the extent of the reduction in trabecular bone parameters in EPO- versus saline-injected mice in each group. All data are mean ± SEM. Data were analyzed by Student’s *t*-test.

To investigate the effect of EPOR signaling in preosteoclasts on bone mass following exogenous EPO administration, we treated both control and Cx3cr1^Cre^ EPOR^f/f^ 12-week-old male and female mice with either EPO or saline for 2 weeks (60 IU injected 3 times per week). This dose was found to have a mild but significant skeletal effect in WT mice [26]. Micro-CT analyses revealed that EPO treatment induced significant bone loss (reduced BV/TV) in both control and Cx3cr1^Cre^ EPOR^f/f^ female mice (Figure 4a). In contrast, EPO treatment caused a less pronounced and not significant reduction in BV/TV in male mice of either genotype, with no significant difference in the effect of EPO between the knockout mice and their controls (16.46% and 19.54% reduction, respectively, Figure 4b).

The extent of the BV/TV reduction induced by EPO in females tended to be lower in the Cx3cr1^Cre^ EPOR^f/f^ mice than in the controls (27.05% vs. 39.26% reduction, respectively, Figure 4a). Although this difference in the response to EPO did not reach statistical significance, it may imply an involvement of EPOR in preosteoclasts in mediating the bone loss induced by EPO.

### 2.5. Low-Dose EPO Does Not Affect Osteoclast Progenitors

**Figure 5:**
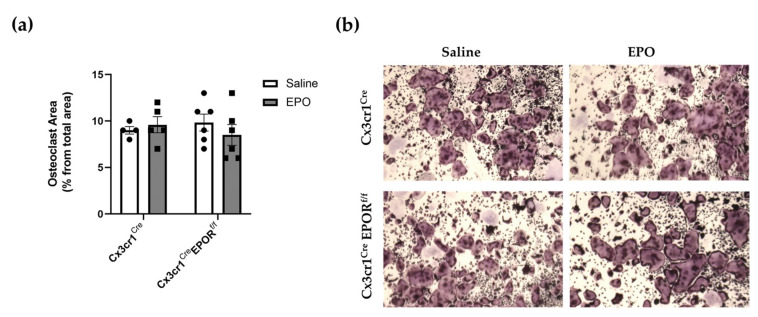
**Low-dose EPO does not increase the number of osteoclast progenitors in vivo.** (**a**) Female Cx3cr1^Cre^EPOR^f/f^ mice and their controls were treated with either saline or EPO (60 IU × 3 per week for 2 weeks). Non-adherent bone marrow cells were grown ex vivo with M-CSF and RANKL, and the total area of multinucleated TRAP^+^ osteoclasts was measured after 6 days as a surrogate for the number of osteoclast precursors in vivo. (**b**) Representative images acquired at ×2 magnification. n = 4–6 in each group. All data are mean ± SEM. *p* values were calculated by 2-way ANOVA.

We have previously reported that high doses of EPO (180 IU × 3 per week for 2 weeks) result in severe bone loss and an increase in the number of osteoclast progenitors in the BM [18,26]. Herein, we used a much lower dose of EPO (60 IU × 3 per week for 2 weeks) in both Cx3cr1^Cre^ EPOR^f/f^ mice and their controls. This regimen still resulted in a significant level of EPO-induced bone loss in female mice (Figure 4a). An ex vivo osteoclastogenesis assay in which a fixed number of BM cells collected from mice in each group were cultured ex vivo in the presence of M-CSF+RANKL was used to assess the number of osteoclasts and thereby, the proportion of osteoclast progenitors in the mice after treatment. The results indicate that low-dose EPO treatment did not affect the number of osteoclast progenitors in the experimental animals or the controls (Figure 5). This suggests that unlike the high-dose treatment, low doses of EPO may stimulate bone resorption via specific effects on osteoclast differentiation [41] but not by increasing the number of preosteoclasts [18,26].

### 2.6. LysM^Cre^ Mediates the Conditional Deletion of EPOR in Preosteoclasts

**Figure 6:**
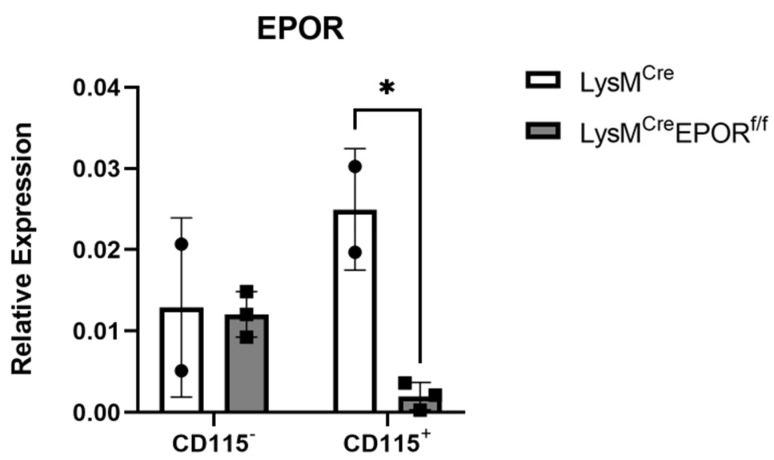
**Confirmation of the conditional deletion of EPOR expression in the monocytic lineage of LysM^Cre^EPOR^f/f^ mice.** EPOR expression, as measured by RT-qPCR in bone-marrow-derived CD115^+^ and CD115^−^ cells from female LysM^Cre^EPOR^f/f^ mice and their controls. Expression of EPOR was normalized to GAPDH. n = 2–3 mice in each group. * *p* < 0.05 versus LysM^Cre^. All data are mean ± SD. *p* values were calculated by 2-way ANOVA.

Because of the non-significant differences in the response to EPO between control and Cx3cr1^Cre^ EPOR^f/f^ mice and the mild skeletal response to low-dose EPO, we employed the well-established LysM^Cre^-induced conditional knockout in the osteoclastic lineage [43,44,45] and increased the administered dose of EPO 3-fold [18,26]. In this model, the levels of EPOR mRNA in preosteoclast (CD115^+^) cells were ~1.9-fold greater in the control LysM^Cre^ mice than in the CD115^−^ cells. Our results also confirm a significant knockout (~12.5-fold) in EPOR mRNA levels in the CD115^+^ cells from the LysM^Cre^EPOR^f/f^ mice compared to their controls, with no change in EPOR expression in the CD115^−^ cells (Figure 6).

### 2.7. The Skeletal Effect of EPO on Both Osteoclast and Osteoblast Precursors Is Partially Mediated by Monocyte EPOR

**Figure 7:**
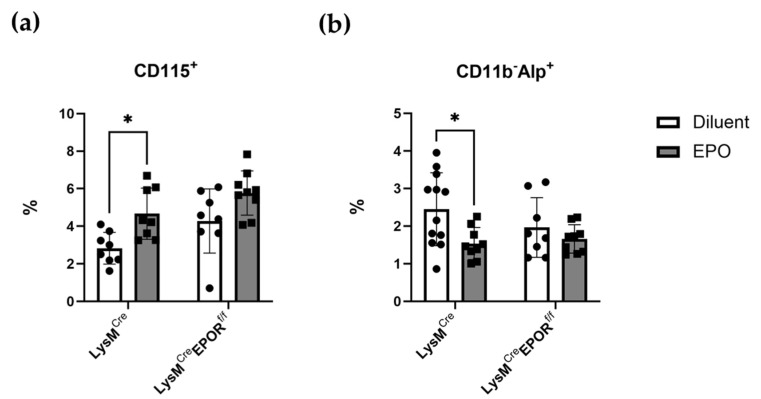
**In vivo effects of monocytic EPOR knockout on bone cell precursors.** Flow cytometry analysis of (**a**) preosteoclasts (CD115^+^) and (**b**) preosteoblasts (CD11b^−^Alp^+^) from the bone marrow of female LysM^Cre^EPOR^f/f^ mice and their controls after treatment with either diluent or EPO. n = 8–12 in each group. * *p* < 0.05 versus diluent. All data are mean ± SEM. *p* values were calculated by 2-way ANOVA.

As the next step, we assessed the effect of EPOR knockout on the percentage of preosteoclasts (CD115^+^) and preosteoblasts (CD11b^−^Alp^+^) in the bone marrow of 12-week-old female C57BL/6J mice after EPO treatment (180 IU × 3/week for 2 weeks). Flow cytometry analysis revealed a 65.06% increase in the number of osteoclast precursors (CD115^+^) and a 37.30% decrease in the number of preosteoblasts (CD11b^−^Alp^+^) in the EPO-treated control mice (Figure 7), which agrees with previous reports [18,26]. Importantly, these effects were abrogated in cells obtained from LysM^Cre^EPOR^f/f^ mice, wherein EPO treatment did not result in any significant changes in preosteoblasts or preosteoclasts.

### 2.8. Conditional Deletion of EPOR in Preosteoclasts Mitigates EPO-Induced Bone Loss in LysM^Cre^EPOR^f/f^ Mice

To investigate the bone density effects of EPOR knockout in preosteoclasts, we injected 12-week-old male and female C57BL/6J mice with EPO (180 IU × 3/week for 2 weeks). At the end of the second week, hemoglobin levels were measured, and the femurs were analyzed by μCT.

**Figure 8:**
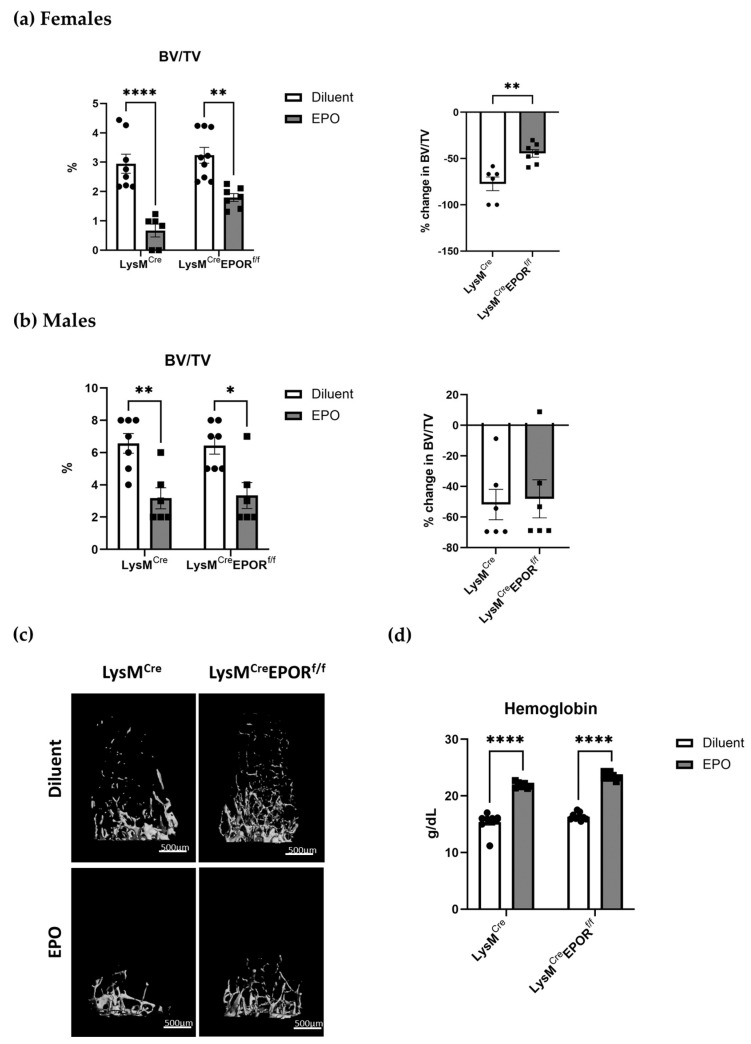
**Monocyte-specific EPOR knockout attenuates EPO induced bone loss.** μCT analysis in the distal femoral metaphysis of diluent- and EPO-injected (180U/inj, 3 times/week for 2 weeks) 12-week-old (**a**) female (n = 6–9 in each group) and (**b**) male (n = 6–7 in each group) transgenic mice carrying a conditional knockout of EPOR in the monocytic lineage LysM^Cre^EPOR^f/f^ mice compared to their LysM^Cre^ controls. *p* values were calculated by 2–way ANOVA in the left panels. Data in the right panels are represented as the extent of reduction in trabecular bone volume/total volume (BV/TV) in EPO- vs. diluent-injected mice in each group and calculated by a Student’s *t*-test. (**c**) Representative 3D μCT images of the distal femur of female mice described in (**a**). (**d**) Hemoglobin levels of EPO- versus diluent-treated LysM^Cre^ or LysM^Cre^EPOR^f/f^ female mice (n = 7–9 in each group). *p* values were calculated by 2-way ANOVA. * *p* < 0.05, ** *p* < 0.01, and **** *p* < 0.0001.

Our results reveal that the conditional deletion of EPOR did not affect the trabecular bone parameters in both males and females (diluent-treated LysM^Cre^ and LysM^Cre^ EPOR^f/f^, Figure 8a,b), suggesting that EPOR signaling in preosteoclasts does not play significant roles under steady-state conditions. EPO treatment significantly lowered the bone density in the trabeculae of both LysM^Cre^ (control) and LysM^Cre^EPOR^f/f^ male and female mice, and in males, the skeletal response to EPO was similar in the control and knockout mice (51.81% and 48.15% reduction, respectively, Figure 8b). However, the extent of bone loss induced by a high EPO dose in female mice with an EPOR deletion in the monocytic lineage (LysM^Cre^EPOR^f/f^) was significantly lower than that in the controls (44.46% versus 77.28% reduction, respectively, *p* = 0.002, Figure 8a). Importantly, despite the dramatic skeletal effects, erythropoiesis was unaffected by the conditional deletion of EPOR, as demonstrated by the EPO-induced increase in hemoglobin (Figure 8d).

## 3. Discussion

We and others have demonstrated consistently that low and high doses of exogenous EPO or transgenic overexpression of EPO lead to severe bone loss accompanied by osteoclast activation and the suppression of bone formation in mouse models [17,18,19,21,22,26]. Interestingly, recent studies demonstrated that this bone loss is independent of the erythropoiesis activities of EPO [17] and is mediated by a non-erythroid cell response. Moreover, the deletion of EPOR in either osteoprogenitors/mature osteoblasts [21,22] or in B cells [40] attenuated EPO-driven bone loss but did not completely block the effect, suggesting that EPOR on other cell lineages also contributes to EPO-mediated bone loss. We previously demonstrated that high doses of EPO target the monocytic lineage by increasing the number of bone marrow preosteoclasts and bone resorption in vivo [18,26,42]. We also showed the direct stimulation of osteoclastogenesis by EPO in vitro [18,26,31], but the question of whether EPO-driven bone loss is mediated by EPOR activation on preosteoclasts in vivo remained elusive. The current study addressed this gap and demonstrated (i) the role of monocytic EPOR in bone metabolism in vivo, in both steady-state conditions and in EPO-treated mice; (ii) while both low and high doses of EPO stimulated osteoclastogenesis in vitro [18,31], only the higher doses led to a dose-dependent increase in osteoclast precursors in vivo (Figure 5 and Figure 7). Our results also showed that the (iii) deletion of EPOR in the monocyte/macrophage lineage does not affect erythropoiesis at steady state and in response to EPO administration (Figure 3 and Figure 8).

We used two mouse lines with a conditional deletion of EPOR in the monocyte/macrophage lineage.

Our results revealed that a low dose of EPO reduced the BV/TV in all animals. There was a tendency towards a more moderate loss in the Cx3cr1^Cre^ EPOR^f/f^ female mice compared to their controls, but this difference was not statistically significant (Figure 4). In addition, there was no difference in the levels of BM osteoclast progenitors in the Cx3cr1^Cre^ EPOR^f/f^ and the controls under EPO treatment and at steady state (Figure 5). It should be noted that a previous study demonstrated that Cx3cr1-deficient mice displayed slight but significant increases in trabecular and cortical thickness and reduced numbers of osteoclasts compared to wild-type mice [50]. These observations motivated us to utilize the better-established LysM^Cre^-line to conditionally knockout EPOR in the osteoclastic lineage [43,44,45] and to increase the dose of EPO [18,26]. Our data revealed that the significant increase in the percentage of BM preosteoclasts (CD115^+^) usually seen after EPO treatment is abrogated in these mice (Figure 7a). Importantly, the bone loss induced by the high EPO dose was significantly attenuated in the LysM^Cre^ EPOR^f/f^ female mice compared to their controls, without interference with the erythropoietic activity of EPO (Figure 8), which calls for further studies on the role of macrophages’ EPOR in context of the erythroblast island niche [53].

Our findings imply that EPOR in the monocytic lineage is responsible, at least in part, for driving the bone mass reduction caused by exogenously administered EPO. While these Cre lines do not discriminate between early precursors and late mature osteoclasts, we previously reported that while EPOR is highly expressed in osteoclast precursors, there is no expression or response to EPO and its analogs by mature osteoclasts [18,42]. It is therefore reasonable to assume that the skeletal response to EPO is partly driven by EPOR in osteoclast precursors.

The discrepancy in the results obtained in the two mice models may be attributed to several factors. One option relates to the limitations arising from the currently available Cre lines that target osteoclast progenitors and may be due to (i) the heterogeneity and plasticity of osteoclast precursors, both under steady state and pathological conditions [54], i.e., not all osteoclast precursors express LysM or Cx3cr1, and (ii) the limited depletion efficiency and targeting specificity in myeloid specific Cre transgenes [55]. These limitations led us to utilize two separate Cre lines, LysM^Cre^ and Cx3cr1^Cre^, with different off-target sites, in order to delete EPOR in the early osteoclast lineage [56]. Cx3cr1^Cre^ is relatively more specific to the monocyte/macrophage lineage than LysM^Cre^, although there are also effects on neutrophils, mast cells, and classical dendritic cells [57]. These Cx3cr1^Cre^ mice have been the focus of much recent skeletal research [48,49,58], since there is evidence that Cx3cr1 is expressed by osteoclast precursors, which facilitates their recruitment to the BM and is downregulated when they differentiate into mature osteoclasts [50,59].

Of note, a recent study reported that, under inflammatory conditions, the expression of Cx3cr1 is maintained in a subpopulation of osteoclasts shown to arise from both Cx3cr1^+^ and Cx3cr1^−^ progenitors [60], suggesting that osteoclast progenitors may have multiple origins depending on their environment. We found that both CD11b^−^CD115^+^Ly6C^hi^ CX3CR1^+^ and CD11b^−^CD115^+^Ly6C^hi^ CX3CR1^−^ populations were elevated (data not shown) in a short-term experiment in which 13-week-old female mice were injected with high doses of EPO. This raises the possibility that CX3CR1^−^ precursors might act in a compensatory manner despite EPOR deletion in the CX3CR1^+^ progenitors, which in turn may mask any differences in the EPO response of Cx3cr1^Cre^ EPOR^f/f^ mice compared to their controls. The apparent heterogeneity in osteoclast progenitors and the limitations of the available models specifically targeting osteoclasts’ precursors present a challenge in defining the role of the osteoclastic EPOR in EPO-induced bone loss. An added complexity is that EPOR expression is detectable only in preosteoclasts and not in the differentiated mature osteoclasts [18,61], so we were unable to use mature osteoclast-specific Cre models, such as Ctsk^cre^ and Trap^cre^. For these reasons, we selected the LysM^Cre^ model to support our conclusions, since, although it lacks specificity for preosteoclasts, LysM is highly expressed in myeloid lineage cells, including monocytes, macrophages, and granulocytes [43].

Of note, while our findings confirm the effective deletion of EPOR in the monocytic lineage of LysM^Cre^ EPOR^f/f^, there is still a possibility of low levels of the non-specific targeting of other immune cells, such as neutrophils. However, the observations that high and low doses of EPO (data not shown), as well as transgenic EPO overexpression [62] had no effect on the percentage of neutrophils in the BM (defined as CD11b^+^Ly6G^+^) and that neutrophils from WT mice barely express EPOR [63] make it more likely that the attenuated EPO-induced bone loss in LysM^Cre^ EPOR^f/f^ mice is due to EPOR deletion in the monocytic lineage.

Interestingly, neither LysM^Cre^ EPOR^f/f^ (Figure 8) nor Cx3cr1^Cre^ EPOR^f/f^ (Figure 1) mice displayed changes in their trabecular bone parameters in steady state compared to their controls, implying that EPOR signaling in the monocytic lineage does not play a significant role under steady state conditions.

EPO-induced bone loss in mice is known to be dose-dependent, wherein low doses of EPO inhibit osteoblast differentiation and mineralization in vitro [17,19], together with a decrease in the percentage of osteoblast precursors in the bone marrow in vivo [26]. In the osteoclast lineage however, while both low and high doses stimulated osteoclastogenesis in vitro (10 mIU/mL and 10 IU/mL, respectively) and in vivo (from 70 IU/week to 540 IU/week), only the higher doses lead to a dose-dependent increase in osteoclast precursors in vivo (Figure 5 vs. [18,26]).

The differential sensitivity of the erythroid cells (Figure 3), compared to osteoclast precursors (Figure 5) in the responses to low-dose EPO, could be explained by the higher EPOR expression in the TER119^+^ erythroid lineage cells compared to CD115^+^ osteoclast lineage cells (Figure S1).

Herein, as reported by two recent studies [21,22], female animals exhibited better protection from EPO-induced bone loss than males. In addition, the sex-differential EPO phenotypes seen in other tissues [64,65,66,67] suggest the involvement of sex hormones. Although no sex-specific differences in the plasma concentration of EPO were detected [68], estrogen has been shown to affect EPO response and mediate gender-specific EPO actions [69]. Future studies on the interaction between estrogen and EPO signaling could elucidate the contribution of sex hormones to the sexually dimorphic features of EPO treatment, particularly in the skeletal system.

The results of this study provide further information about the complex role of EPO in skeletal biology. EPOR in B cells promotes bone loss via the upregulation of osteoclastogenic signals and by inducing their transdifferentiation into osteoclasts in the presence of therapeutic doses of EPO [40]. EPOR on the osteoblast lineage mainly mediates the physiological skeletal effects of EPO, with a less noticeable contribution in response to EPO treatment [21]. We can now conclude that, while EPOR in the osteoclast lineage does not have a significant role during steady-state bone homeostasis, it drives at least part of the bone loss induced by high-dose EPO treatment.

Overall, our study provides evidence that the EPO/EPOR axis in the monocytic lineage has a direct role in EPO-induced bone loss. Further in-depth characterization of the identity of the osteoclast precursor subsets and their osteoclastogenic capacity will shed more light on the cells directly targeted by EPO and their relative contribution to EPO-driven skeletal effects, and taking into consideration the dose-dependent nature of the EPO-induced bone loss. Since recombinant erythropoietin is widely used in clinical practice to treat anemia associated with chronic kidney disease in patients who already suffer from compromised bone health, it is advisable to monitor these patients closely, and to minimize the potential adverse skeletal outcomes by administering the lowest effective dose of EPO for the shortest possible time [26]. Combining EPO with other anti-resorptive agents might also prove advantageous [42]. In addition, these findings should motivate the search for new alternatives to erythropoietin therapy, such as hypoxia-inducible factor-prolyl hydroxylase inhibitors. The ideal agent would stimulate erythropoiesis without the EPO-associated bone loss [70].

## 4. Materials and Methods

### 4.1. Materials

Alpha-MEM and fetal bovine serum (FBS) were purchased from Rhenium (Modiin, Israel), culture plates were from Corning (New York, NY, USA). As a source of M-CSF, we used supernatant from CMG 14-12 cells, containing 1.3 μg/mL M-CSF [18,71]. RANKL was purchased from R&D Systems, Minneapolis, MN, USA. Erythropoietin (EPO) used in the study was obtained from GMP-manufactured sterile syringes containing rHuEPO (Epoetin alfa, Eprex^®^, Janssen) as used for patient care. These were kindly provided by Janssen Cilag, Israel.

### 4.2. Animals

Mouse handling and all experimental procedures were approved by the Institutional Animal Care and Use Committee of the Tel-Aviv University (permit numbers: 01-19-032, M-14-093) and were performed in accordance with the approved guidelines. All transgenic mice were kept in a specific pathogen free (SPF) facility and were of the C57BL/6J genetic background. Experiments were performed with male and female mice between the ages of 11 and 12 weeks; Cx3cr1^Cre^EPOR^f/f^ mice and LysM^Cre^EPOR^f/f^ mice and their corresponding controls. Cx3cr1^Cre^ mice were kindly provided by Prof. Steffen Jung, the Weizmann Institute of Science, Rehovot, Israel and maintained in the animal facility of the Tel-Aviv university. Both mouse lines were crossed with EPOR^f/f^ mice in our facility. Both Cre lines are homozygous.

### 4.3. Cell Culture

#### 4.3.1. Isolation and Culture of Bone Marrow-Derived Monocytes

Murine monocytes were harvested from femurs and tibias of 12-week-old male and female transgenic mice (Cx3cr1^Cre^EPOR^f/f^ mice and their corresponding controls Cx3cr1^Cre^ ) by negative selection using a monocyte isolation kit from Miltenyi Biotec (#130-100-629; Auburn, CA, USA). Isolated monocytes were seeded on non–tissue culture-treated plates in α-MEM containing 10% FBS, supplemented with 100 ng/mL M-CSF (in the form of 10% v/v culture supernatant from CMG 14–12 cells) and cultured for 4 days to induce differentiation into bone marrow derived macrophages (BMDM) [72]. BMDM were used to extract RNA for real-time PCR or for in vitro osteoclastogenesis

#### 4.3.2. Isolation of CD115^+^ Monocytes

Bone marrow CD115^+^ monocytes were sorted by a CD115 microbead kit from Miltenyi Biotec (#130-096-354, Bergisch Gladbach, Germany) according to the manufacturer’s instructions. CD115^+^ cells were used to extract RNA for real-time PCR.

In vitro osteoclastogenesis: BMDM obtained as described above were plated in 96-well plates (8000 cells per well) with standard medium supplemented with 20 ng/mL M-CSF (in the form of 2% *v*/*v* culture supernatant from CMG 14–12 cells) and 50 ng/mL RANKL (R&D Systems, Minneapolis, MN, USA), which was replaced every 2 days. On the 4–5th day, multinucleated osteoclasts were stained using a tartrate-resistant acid phosphatase (TRAP) kit (Sigma-Aldrich, St. Louis, MO, United States), and the relative TRAP-positive surface was measured using ImageJ software.

Ex vivo osteoclastogenesis: Bone marrow cells were harvested from femurs and tibias of 12-week-old female mice injected with either EPO or saline for 2 weeks (60 IUx 3 times per week) and were then seeded into tissue-culture-treated plates in standard medium and allowed to attach overnight. Non-adherent cells were plated into 96-well plates in standard medium supplemented with M-CSF (in the form of 2% *v*/*v* culture supernatant from CMG 14–12 cells) and 50 ng/mL recombinant murine RANKL. The medium was replaced every other day. After 6 days, the cells were stained for tartrate-resistant acid phosphatase (TRAP), and the relative TRAP-positive surface was measured using ImageJ software(1.53 s, NIH, Bethesda, Maryland, USA).

### 4.4. Microcomputed Tomography (μCT)

Femurs (one per mouse) were examined using the μCT50 system (Scanco Medical AG, Bruttisellen, Switzerland) [73,74]. Briefly, scans were performed with a 10 μm resolution, 90 kV energy, 114 mA intensity, and 1100 ms integration time. The mineralized tissues were segmented by a global thresholding procedure following the Gaussian filtration of the stacked tomographic images [75]. Trabecular bone parameters were measured in the secondary spongiosa of the distal femoral metaphysis.

### 4.5. Hemoglobin Levels

Hemoglobin (Hgb) levels were measured in venous blood (drawn from the facial vein) by means of a “Mission Plus” hemoglobin/hematocrit meter (Acon, San Diego, CA, USA).

### 4.6. Flow Cytometry

Bone marrow (BM) cells were flushed from femurs or tibias, and red blood cells were lysed using ACK lysis buffer (Quality Biological, Gaithersburg, MD, USA), except for experiments with Anti-Ter119^+^ staining. The cells were then stained for 20 min at 4 °C with conjugated anti-mouse antibodies (see Table 1 for a list of the antibodies used). After this time, the cells were washed with PBS containing 1% FBS and analyzed by either Gallios or Cytoflex flow cytometers and Kaluza or CytExpert software (all from Beckman Coulter, Indianapolis, IN, USA).

### 4.7. Real-Time RT PCR

Total RNA was extracted using the TriRNA Pure kit (Cat.# TRPD200, Geneaid, New Taipei City, Taiwan), and cDNA was synthesized using the qScript cDNA synthesis kit (Quantabio, MA, USA). “Real-time” quantitative PCR (RT-qPCR) was performed on a StepOnePlus instrument using the SYBR Green reagent (both from Applied Biosystems, CA, USA). Relative gene expression was calculated using the ΔΔCT method following normalization to the expression of HPRT or GAPDH as housekeeping genes. Primers used for PCR were as follows: (F, forward; R, reverse): EPOR,F;GTCCTCATCTCGCTGTTGCT EPOR,R; ATGCCAGGCCAGATCTTCT; HPRT,F TCCTCCTCAGACCGCTTTT; HPRT,R CCTGGTTCATCATCGCTAATC; GAPDH,F ACCCAGAAGACTGTGGATGG; GAPDH,R CACATTGGGGGTAGGAACAC.

### 4.8. Statistical Analysis

Values are expressed as mean ± SEM (standard error of the mean) unless otherwise indicated. A Student’s *t*-test was used for calculating statistical significance when comparing two groups of variables. In experiments with >2 groups of variables, either 1-way or 2-way ANOVA was applied. The level of statistical significance was set at *p* < 0.05. Asterisks between bars indicate significant differences between two groups (* *p* < 0.05, ** *p* < 0.01, *** *p* < 0.001, and **** *p* < 0.0001). All statistical analyses were performed using Prism 9 (GraphPad).

## Figures and Tables

**Table 1:** Antibodies used for flow cytometry analysis.

Antibody	Source	Identifier
TER-119-APC	BioLegend	Cat#: 116211
CD115-APC	eBioscience	Cat#: 14115282
F4/80-APC	BioLegend	Cat#: 123115
CD11b-APC	BioLegend	Cat#: 101211
CD115-PE	Miltenyi Biotec	Cat#: 130112828
LY6C-PerCP/Cy5.5	BioLegend	Cat#: 128011
Alkaline Phosphatase (ALPL)	R&D systems	Cat#: AF2910
Goat IgG (H+L)-PE	R&D systems	Cat#: F0107
Anti N-terminus mEPOR	[76,77]	
Donkey anti-rabbit IgG H&L-Alexa Fluor^®^ 488	abcam	Cat#: ab150073

## Supplementary Materials

The following supporting information can be downloaded at: https://www.mdpi.com/article/10.3390/ijms231912051/s1.
