# Supplementary material for: Erythropoietin Receptor (EPOR) Signaling in the Osteoclast Lineage Contributes to EPO-Induced Bone Loss in Mice
Source: Int J Mol Sci. 2022 Oct 10;23(19):12051. doi: 10.3390/ijms231912051 (PMC9570419; doi:10.3390/ijms231912051)
Supplement: Supplementary file 1 [file ijms-23-12051-s001.zip › ijms-1890963-supplementary.pptx]

## Slide 1
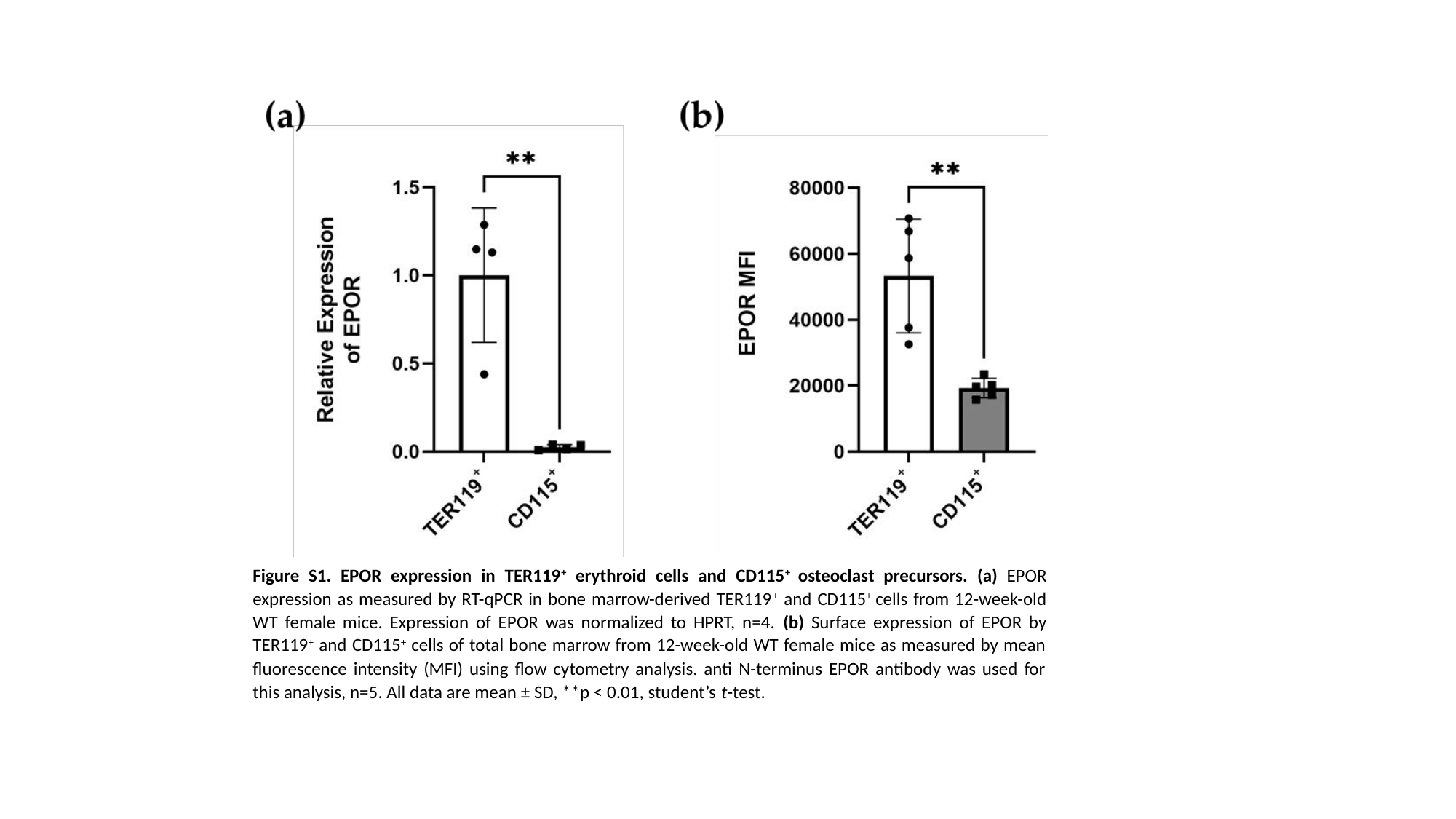

Figure S1. EPOR expression in TER119+ erythroid cells and CD115+ osteoclast precursors. (a) EPOR expression as measured by RT-qPCR in bone marrow-derived TER119+ and CD115+ cells from 12-week-old WT female mice. Expression of EPOR was normalized to HPRT, n=4. (b) Surface expression of EPOR by TER119+ and CD115+ cells of total bone marrow from 12-week-old WT female mice as measured by mean fluorescence intensity (MFI) using flow cytometry analysis. anti N-terminus EPOR antibody was used for this analysis, n=5. All data are mean ± SD, **p < 0.01, student’s t-test.
